# Lactoferrin Modulates Radiation Response Under Hypoxic Conditions, Possibly Through the Regulation of ROS Production in a Cell Type-Specific Manner

**DOI:** 10.3390/antiox14010001

**Published:** 2024-12-24

**Authors:** Daitoku Murakami, Takahiro Fukazawa, Michihito Kyo, Mutsumi Miyauchi, Shigehiro Ono, Tomonao Aikawa, Nobuyuki Hirohashi, Keiji Tanimoto

**Affiliations:** 1 Department of Radiation Disaster Medicine, Research Institute for Radiation Biology and Medicine, Hiroshima University, Hiroshima 734-8553, Japan; d-murakami@hiroshima-u.ac.jp (D.M.); mkyo@hiroshima-u.ac.jp (M.K.); hirohasi@hiroshima-u.ac.jp (N.H.); 2 Department of Oral and Maxillofacial Surgery, Graduate School of Biomedical and Health Sciences, Hiroshima University, Hiroshima 734-8553, Japan; onoshige@hiroshima-u.ac.jp (S.O.); aikawat@hiroshima-u.ac.jp (T.A.); 3 Division of Medical Research Support, Advanced Research Support Center, Ehime University, Toon 791-0295, Japan; fukazawa.takahiro.ds@ehime-u.ac.jp; 4 Department of Oral and Maxillofacial Pathobiology, Graduate School of Biomedical and Health Sciences, Hiroshima University, Hiroshima 734-8553, Japan; mmiya@hiroshima-u.ac.jp

**Keywords:** lactoferrin, hypoxia, radiation, reactive oxygen species (ROS)

## Abstract

Lactoferrin (LF) is an iron-binding glycoprotein of the transferrin family and has been suggested to have a variety of biological functions, including anticancer activity. However, the effects of LF and its mechanisms in anticancer therapies, especially in radiotherapy against cancer cells under hypoxic conditions, are not well-determined. In this study, we focused on the molecular mechanisms of LF functions in cells under hypoxic conditions. High-dose LF treatment showed cytotoxic activity in a variety of cells, including both non-cancer and cancer cells. Interestingly, hypoxic treatment increased the sensitivity to LF in some cancer cells but decreased it in non-cancer cells. LF treatment also altered sensitivity to radiation treatment: LF significantly increased the viability of irradiated KD non-cancer cells under hypoxic conditions but decreased that of HSC2 cancer cells. These effects were only observed when LF was treated within 3 h of irradiation, but not before irradiation. Importantly, knockdown of *HIF1A* counteracted these effects in both cell lines. Measurements of ROS activity showed that LF decreased ROS production in KD cells but increased it in HSC2 cells, resulting in a decrease in γH2AX foci in KD cells but an increase in HSC2 cells. RNA-seq and gene set enrichment analysis showed that LF treatment regulated gene expression related to the cell cycle, apoptosis, inflammation, and the NRF2 antioxidant signaling pathway. Quantitative RT-PCR confirmed the downregulation of the pro-apoptotic gene *ASC* in KD cells and the NRF2-regulated genes in HSC2 cells by LF treatment. Knockdown experiments confirmed the role of ASC in irradiated KD cells and NRF2 in irradiated HSC2 cells with LF treatment. In conclusion, lactoferrin was shown to affect radiation treatment by regulating apoptosis and NRF2 signaling in a cell type-specific manner under hypoxic conditions, suggesting its potential application as a protector or sensitizer for radiation therapy.

## 1. Introduction

Oral cancer is a cancer that will affect approximately 390,000 individuals worldwide and cause 190,000 deaths in 2022 [1]. Histologically, more than 90% of oral cancers are squamous-cell carcinomas (SCC), which originate from epithelial cells [2]. Treatments for oral cancer consist primarily of surgery, radiotherapy, and chemotherapy. Despite advances in the treatment of oral cancer, the current 5-year survival rate remains around 50% [3,4]. Therefore, improvements in effective treatments and/or drugs are desired.

Hypoxia, a condition characterized by low oxygen levels, is a common feature of the tumor microenvironment and is known to induce resistance to radiotherapy [5]. Hypoxic cells exhibit altered cellular metabolism and reduced production of reactive oxygen species (ROS) upon radiation exposure, resulting in decreased DNA damage and increased cancer cell survival [6]. Hypoxia-inducible factors (HIFs) are key transcription factors in a hypoxic microenvironment that control tumor proliferation, angiogenesis, invasion, and therapy resistance through the regulation of target gene expression [7,8,9,10]. Thus, hypoxia or hypoxic signals are recognized as potential targets to improve current therapies, including chemo-radiation therapy.

Lactoferrin (LF), a member of the transferrin family and an 80 kDa iron-binding glycoprotein found in various secretory fluids such as milk, saliva, and tears, has attracted attention for its diverse biological functions, including antimicrobial, anti-inflammatory, and immunomodulatory activities [11,12,13,14,15]. Recent studies have suggested that lactoferrin may also play an important role in modulating the cellular response to radiotherapy, a critical component of cancer treatment [16,17]. Bovine lactoferrin (bLF) is readily available and has been designated and approved by the United States Food and Drug Administration [18]. Although bLF has been suggested to have anticancer effects in lung, breast, and prostate cancer in vitro [19,20,21] and in non-small-cell lung cancer (NSCLC) and renal cell cancer (RCC) in clinical trials [22,23,24,25,26], the mechanism remains unclear. Emerging evidence has highlighted a potential link between lactoferrin and ferroptosis, an iron-dependent form of regulated cell death characterized by excessive lipid peroxidation and redox imbalance [27,28]. Ferroptosis is increasingly recognized as a critical driver in numerous pathological processes, including neurodegeneration, ischemia-reperfusion injury, and cancer progression. Given its unique ability to chelate free iron and suppress the generation of reactive oxygen species, lactoferrin has been hypothesized to act as a natural inhibitor of ferroptosis, providing a protective mechanism against iron-induced cellular damage. Elucidation of the molecular crosstalk between lactoferrin and ferroptosis may provide transformative insights into the development of innovative therapies targeting iron dysregulation and oxidative stress in human diseases. Indeed, we have previously reported that hypoxia differentially regulates the cytotoxic activities of LF in different cell lines, possibly through the p53 signaling pathway [29]. It has also been suggested that LF regulates ferroptosis through the expression of *ACSL4*, although further studies including comparative analyses are needed to reach a definitive conclusion. Understanding the mechanisms by which lactoferrin influences the radiation response under hypoxic conditions is crucial for improving the efficacy of radiotherapy in solid clinical tumors containing hypoxic cancer cells.

In this study, we focused on the modulation activities of lactoferrin on radiation responses in cells under hypoxic conditions and demonstrated its cell-type-specific activities, possibly through the regulation of reactive oxygen species (ROS) production. These findings provide new insights into the clinical application of lactoferrin to develop more effective and safer novel chemo-radiotherapy.

## 2. Materials and Methods

### 2.1. Chemicals

All chemicals were of analytical grade and were purchased from FUJIFILM Wako Pure Chemicals (Osaka, Japan), Sigma-Aldrich (St. Louis, MO, USA).

### 2.2. Cell Culture and Treatments

Human cell lines, TIG-3 (lung fibroblast), HEK293 (human embryonic kidney cells), KD (lip fibroblast), HSC2, HSC4, and Ca9-22 (oral squamous-cell carcinoma), were purchased from the Japanese Cancer Research Resource Bank (JCRB) and have been maintained as original stocks. Cells were normally maintained in DMEM (NACALAI TESQUE, Inc., Kyoto, Japan) containing 10% fetal bovine serum (FBS; BioWhittaker, Verviers, Belgium), and were used within 3 months of passage from the original stocks. Cell proliferation capacity was evaluated by a conventional MTT dye reduction assay [30]. Briefly, 4 × 10^3^ cells were seeded on a 96-well plate and cultured with or without LF for 1 day, after irradiated cells were treated with or without LF for 72 h under normoxic (21% O_2_) or hypoxic (1% O_2_ in a hypoxic chamber) conditions. After incubation under the experimental treatment, 10 μL of 0.4% MTT reagent and 0.1 M sodium succinate were added to each well, and after 2 h of incubation, 150 μL of DMSO was added to dissolve the purple formazan precipitate. The formazan dye was measured spectrophotometrically (570–650 nm) using a MAXline^®^ Microplate Reader (Molecular Devices Corp., Sunnyvale, CA, USA). The drug sensitivity of each treatment was assessed by comparing the ratio of the treatment group to the control group.

For expression analyses, cells (1 × 10^6^ cells/10 cm diameter dish) were treated with or without the indicated concentration of LF and subsequently cultured under normoxic or hypoxic conditions for 24 h. They were then harvested by centrifugation and cell pellets were stored at −80 °C until use.

The indicated doses of ^137^Cs γ-ray irradiation were applied using a Gammacell 40 Exactor (MDS Nordion, Ottawa, ON, Canada) under normoxic conditions, and irradiated cells were further incubated until the indicated time points. Cell proliferation capacity was then assessed using the MTT assay as described above.

For knockdown experiments, KD, HSC2, Ca9-22, or HSC4 cells were transfected with siRNA for non-specific (siNS, No. 1027310), *HIF1A* (siHIF1A, SI02664053), *PYCARD* (siASC, SI02639812), or *NFE2L2* (siNRF2, SI03246614) (QIAGEN, Inc., Valencia, CA, USA) by using Lipofectamine^TM^ RNAiMAX (Invitrogen, Carlsbad, CA, USA) for 24 h, and then cells were irradiated and treated with or without LF under normoxic or hypoxic conditions for 72 h. Cell proliferation capacity was evaluated with the MTT assay described above.

### 2.3. RNA Preparation and Quantitative RT-PCR Analysis

Total RNA was prepared from frozen cell pellets using NucleoSpin^®^ RNA (MACHEREY-NAGEL GmbH&Co., KG, Düren, Germany) according to the manufacturer’s instructions. To avoid genomic detection by RT-PCR, all RNA samples were treated with DNase, which was included in the kit. Then, 1 μg of total RNA was reverse-transcribed using a ReverTra Ace^®^ qPCR RT Kit (TOYOBO, Osaka, Japan). qPCR analysis was performed with cDNA from each cell using the THUNDERBIRD Next SYBR qPCR Mix (TOYOBO) according to the manufacturer’s protocol. *ACTB* was used as an internal control. PCR reactions were performed using a 7500 real-time PCR system (Applied Biosystems, Foster City, CA, USA) under standard conditions. Relative expression was calculated by using *ACTB* expression as the denominator for each cell line.

### 2.4. Immunoblot Analysis

For the analysis of protein expression, whole cell extracts were prepared from cultured cells as previously described [31]. Briefly, 40 μg of extracts were blotted onto PVDF filters after SDS-polyacrylamide gel electrophoresis. Anti-β-actin (A5441, Sigma) and anti-caspase 8 (#9746, Cell Signaling TECHNOLOGY, Danvers, MA, USA) were used as primary antibodies at 1:5000 and 1:500 dilutions, respectively. A 1:2000 dilution of anti-mouse IgG horseradish peroxidase conjugate (#7076, #7074, CST) was used as a secondary antibody. Immunocomplexes were visualized by using the enhanced chemiluminescence reagent SuperSignal West Pico PLUS (Thermo Fisher Scientific K.K., Tokyo, Japan).

### 2.5. DCFH-DA/H2DCFDA Cellular ROS Assay

DCFH-DA was used to assess intracellular reactive oxygen species. KD and HSC2 cells (4 × 10^3^ cells) were seeded on a 96-well plate and cultured for 1 day. The cells were then exposed to 5 or 10 Gy of ^137^Cs γ-ray and incubated with or without LF for 24 h under hypoxic conditions. After incubation, the cells were stained with DCFH-DA solution (ROS Assay Kit-Highly Sensitive DCFH-DA; Dojindo Laboratories, Kumamoto, Japan) according to the manufacturer’s instructions, and multiple images were captured using a BZ-8000 microscope (KEYENCE, Osaka, Japan). Positive cells per field were counted and calculated as ROS production in each cell.

### 2.6. Immunofluorescence Analysis

KD or HSC2 cells grown on coverslips were irradiated with the indicated doses of ^137^Cs γ-rays under normoxic conditions, and the irradiated cells were further incubated for 24 h under hypoxic conditions. Cells on coverslips were then fixed with 4% paraformaldehyde, permeabilized with 0.5% Triton X-100, and stained with anti-γH2AX (1:1000) as a primary antibody. Rhodamine-conjugated sheep anti-mouse immunoglobulin G (IgG) (1:1000) (Chemicon, Temecula, CA, USA) was used as the secondary antibody. After several washes, nuclei were stained with 4–6-diamidino-2-phenylindole (DAPI). The subnuclear distribution of γH2AX foci was observed, and images were captured using a fluorescence BZ-8000 microscope. For each condition, γH2AX foci per field were counted, and the mean values were calculated by the authors.

### 2.7. RNA-Seq Analysis

RNA-seq analysis was performed by Novogene Co., Ltd. (Tianjin, China) according to the standard analysis flow as previously described [32]. Briefly, mRNA was purified from total RNA using poly-T oligo-attached magnetic beads. After fragmentation, first-strand cDNA was synthesized using random hexamer primers, followed by second-strand cDNA synthesis using dUTP for the directional library. The library was verified using Qubit and real-time PCR for quantification and Bioanalyzer for size distribution detection. Quantified libraries were pooled and sequenced on DNBSEQ-T7 platforms according to effective library concentrations and data volume. Raw data (raw reads) in fastq format were first processed by in-house Perl scripts. In this step, clean reads were obtained by removing reads containing poly-N and low-quality reads from the raw data. All downstream analyses were based on clean, high-quality data. The reference genome and gene model annotation files were downloaded directly from the genome website. The index of the reference genome was created using Hisat2 v2.0.5, and paired-end clean 1 reads were aligned to the reference genome using Hisat2 v2.0.5 [33]. FeatureCounts v1.5.0-p3 was used to count the number of reads mapped to each gene [34]. The FPKM of each gene was then calculated based on the length of the gene and the number of reads mapped to that gene. Prior to differential gene expression analysis, the read counts for each sequenced library were scaled by a scaling normalization factor using the edgeR program package. Differential expression analysis of two conditions was performed using the edgeR R package (3.22.5) [35]. *p*-values were corrected using the Benjamini and Hochberg method. A corrected *p*-value of 0.05 and an absolute fold change of 2 were used as thresholds for significant differential expression. Gene set enrichment analysis was performed on the identified genes using Metascape (https://metascape.org/gp/index.html#/main/step1, accessed on 13 May 2024). Overlapping genes were extracted using jvenn, a plug-in for the jQuery javascript library (https://jvenn.toulouse.inrae.fr/app/example.html; accessed on 13 May 2024). These RNA sequence data were deposited in DDBJ DRA (BioProject No.: PRJDB19732).

### 2.8. Statistical Analysis

Statistical tests were performed using EZ-R version 1.55. To compare time groups, data were analyzed using *t*-tests. When more than three groups were compared, data were analyzed with a one-way ANOVA test, and the Tukey–Kramer method was used for post hoc group comparisons. *p* ≤ 0.05 was considered statistically significant [36].

## 3. Results

### 3.1. Cytotoxic Activities of Lactoferrin in Different Cell Lines

**Figure 1:**
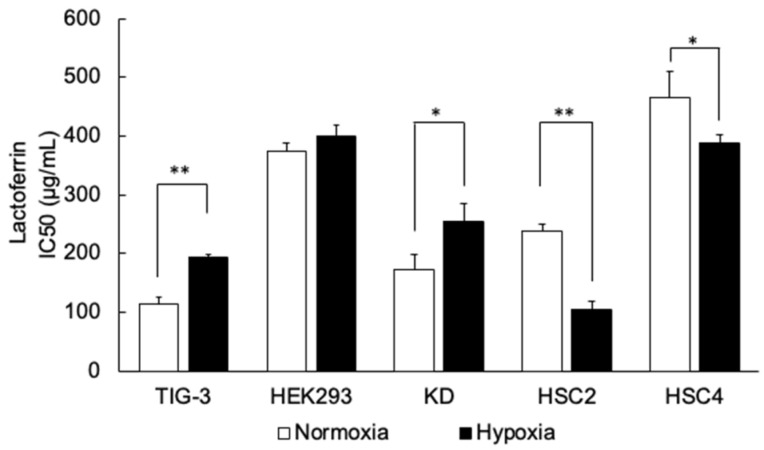
Cytotoxic activities of lactoferrin in different cell lines under normoxic or hypoxic conditions. Cytotoxic activities in cell lines under normoxic (21% O_2_) or hypoxic (1% O_2_) conditions were evaluated as IC_50_ values using the MTT assay. Values are mean and SD (*n* = 3). Statistical significance is indicated by * *p* < 0.05, ** *p* < 0.01 (paired samples indicated).

To clarify whether lactoferrin (LF) has cytotoxic activities in various cell lines under both normoxic and hypoxic conditions, cellular viability with LF treatment was first evaluated using the MTT assay (Figure 1). As a result, LF showed cytotoxic activities in most of the tested cell lines with a wide range of IC_50_ values among cell lines, e.g., from 100 μg/mL in TIG-3 lung fibroblast cells to more than 1000 μg/mL in Ca9-22 oral cancer cells at the highest experimental dose. Interestingly, hypoxic incubation altered the sensitivity to LF in some cell lines. Hypoxia decreased sensitivity to LF (increased IC_50_ values) in TIG-3 and KD (non-cancer fibroblast cells) and increased sensitivity (decreased IC_50_ values) in HSC2 and HSC4 (oral cancer cells). Therefore, KD and HSC2 cells were the focus of the following experiments.

### 3.2. Effects of Lactoferrin on the Radiation Response of Cells Under Hypoxic Conditions

**Figure 2:**
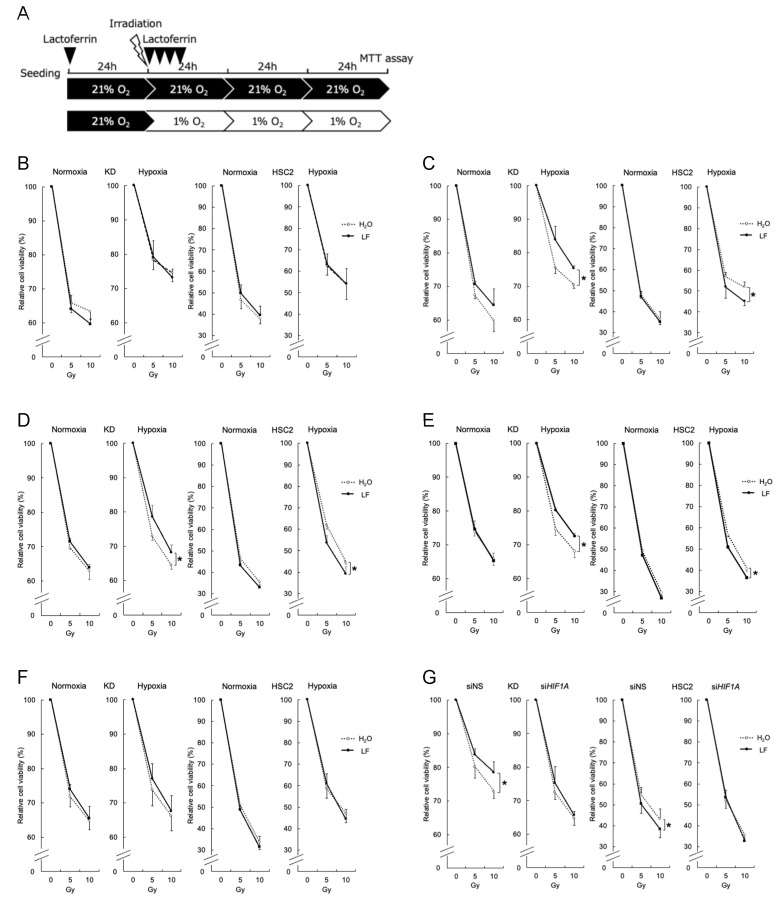
Effects of lactoferrin on radiation responses under normoxic or hypoxic conditions. (**A**) The experimental schedule is shown. Cells were cultured under normoxic (normoxia: 21% O_2_) or hypoxic (hypoxia: 1% O_2_) conditions for the indicated time periods. All cells were in normoxia when cells were irradiated. Lactoferrin was added to the cell culture medium at (**B**) 24 h before, (**C**) 0, (**D**) 1, (**E**) 2, or (**F**) 3 h after irradiation as indicated by arrowheads. After further incubation under normoxic or hypoxic conditions for 72 h, the relative cell viability of KD and HSC2 cells after γ-ray irradiation (0, 5, or 10 Gy) was evaluated using the MTT assay. (**G**) Cells were transfected with non-specific control (siNS) or siHIF1A 24 h after seeding and further incubated for 24 h before irradiation. Lactoferrin was added to the cell culture medium immediately after irradiation. After further incubation under hypoxic conditions for 72 h, the relative cell viability of KD and HSC2 cells after γ-ray irradiation (0, 5, or 10 Gy) was evaluated using the MTT assay. Values are mean and SD (*n* = 3). Statistical significance is indicated by * *p* < 0.05.

To determine the influence of LF treatment on radiation responses in cell lines, a low dose of LF (10 μg/mL) was treated at different time points: 24 h before (pretreatment) or 0, 1, 2, and 3 h after irradiation (Figure 2A). Cells were further incubated under normoxic or hypoxic conditions for 72 h, and cell viability was assessed using the MTT assay. Pretreatment with LF did not affect cell viability after irradiation under both normoxic and hypoxic conditions (Figure 2B). However, when LF was treated immediately after irradiation, the viability of KD cells increased, while that of HSC2 cells decreased under hypoxic conditions (Figure 2C). The cell viability of KD cells under normoxic conditions appeared to increase with LF treatment, but this was not significant. Similar effects of LF on KD and HSC2 cells were observed when LF treatment was applied 1 or 2 h after irradiation (Figure 2D,E), but these effects disappeared when LF treatment was applied 3 h after irradiation (Figure 2F). Importantly, knockdown of the *HIF1A* gene, the master regulator of hypoxia-inducible transcription, eliminated the above effects of LF in both cell lines, suggesting its significant role in LF activity under hypoxic conditions (Figure 2G). Similar results were also observed in Ca9-22 and HSC4 cells (Figure S1).

### 3.3. Effects of Lactoferrin on Radiation-Induced Cell Damage Under Hypoxic Conditions

**Figure 3:**
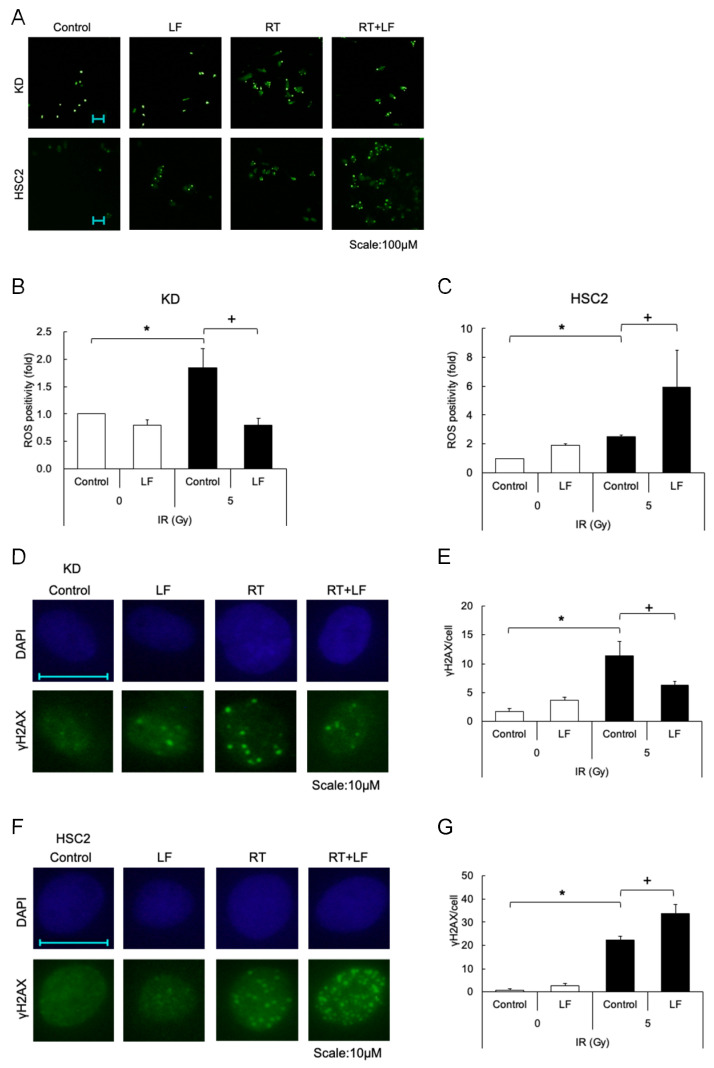
Effects of lactoferrin on radiation-induced cell damage under hypoxic conditions. (**A**) KD or HSC2 cells (4 × 10^3^ cells) were seeded on a 96-well plate. After 24 h culture, cells were exposed to 5 Gy of ^137^Cs γ-rays and incubated with or without lactoferrin (10 μg/mL) for 24 h under hypoxic conditions. Reactive oxygen species (ROS) were evaluated using the DCFH-DA/H2DCFDA cellular ROS assay according to the manufacturer’s instructions and multiple images were captured using a BZ-8000 microscope. Positive cells per field were counted and calculated as relative ROS production in (**B**) KD and (**C**) HSC2 cells. Control indicates untreated cells. (**D**,**E**) KD or (**F**,**G**) HSC2 cells grown on coverslips were irradiated with 5 Gy of ^137^Cs γ-rays under normoxic conditions and further incubated with or without lactoferrin (10 μg/mL) for 24 h under hypoxic conditions. Cells on coverslips were then fixed and stained with anti-γH2AX. Cell nuclei were stained with DAPI. Multiple images were captured using a BZ-8000 microscope, and γH2AX foci per nucleus were counted and averaged. Control indicates untreated cells. Values are mean and SD (*n* = 3). Statistical significance is indicated by ^+^ *p* < 0.05 (Control vs. LF), * *p* < 0.05 (paired samples indicated).

To clarify the mechanisms behind the protective effects of LF in KD cells and sensitizing effects in HSC2 cells after irradiation under hypoxic conditions, radiation-induced reactive oxygen species (ROS) and DNA double-strand breaks (DSBs) were evaluated using the DCFH-DA/H2DCFDA cellular ROS assay and immunofluorescence analysis. A relatively low dose of LF (10 μg/mL) was applied immediately after irradiation, and the cells were further cultured under hypoxic conditions for 24 h. The ROS assay showed that the intracellular levels of ROS increased in both cells after irradiation (Figure 3A–C). LF treatment significantly decreased ROS levels in KD cells but increased them in HSC2 cells (Figure 3A–C). LF treatment alone also slightly increased ROS in HSC2 cells. The immunofluorescence analysis revealed that nuclear γH2AX foci significantly increased in both cells after irradiation; however, LF treatment significantly decreased these foci in KD cells and increased them in HSC2 cells (Figure 3D–G). LF treatment alone also appeared to increase ROS in both cells.

### 3.4. Lactoferrin Regulates Multiple Signaling Pathways, Including Apoptosis and NRF2 Antioxidant Responses

**Figure 4:**
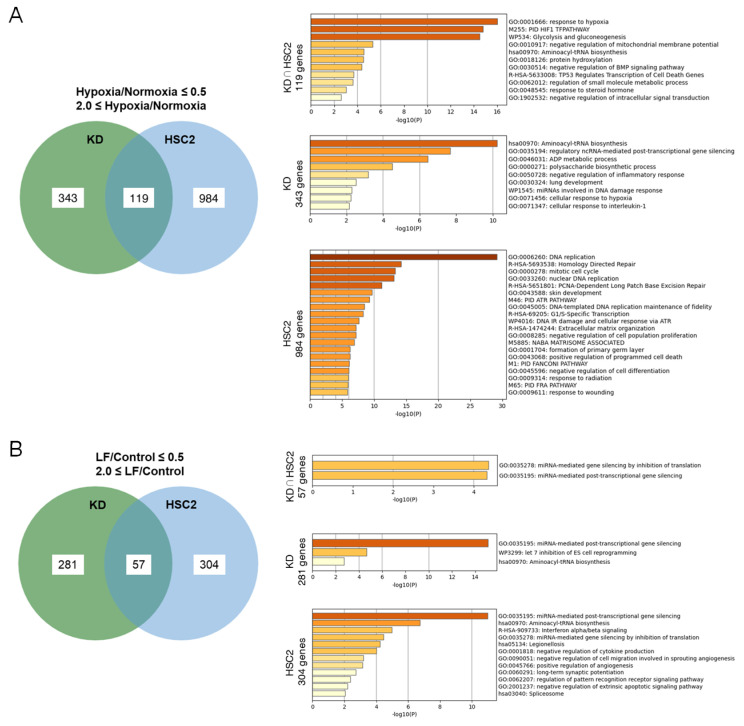

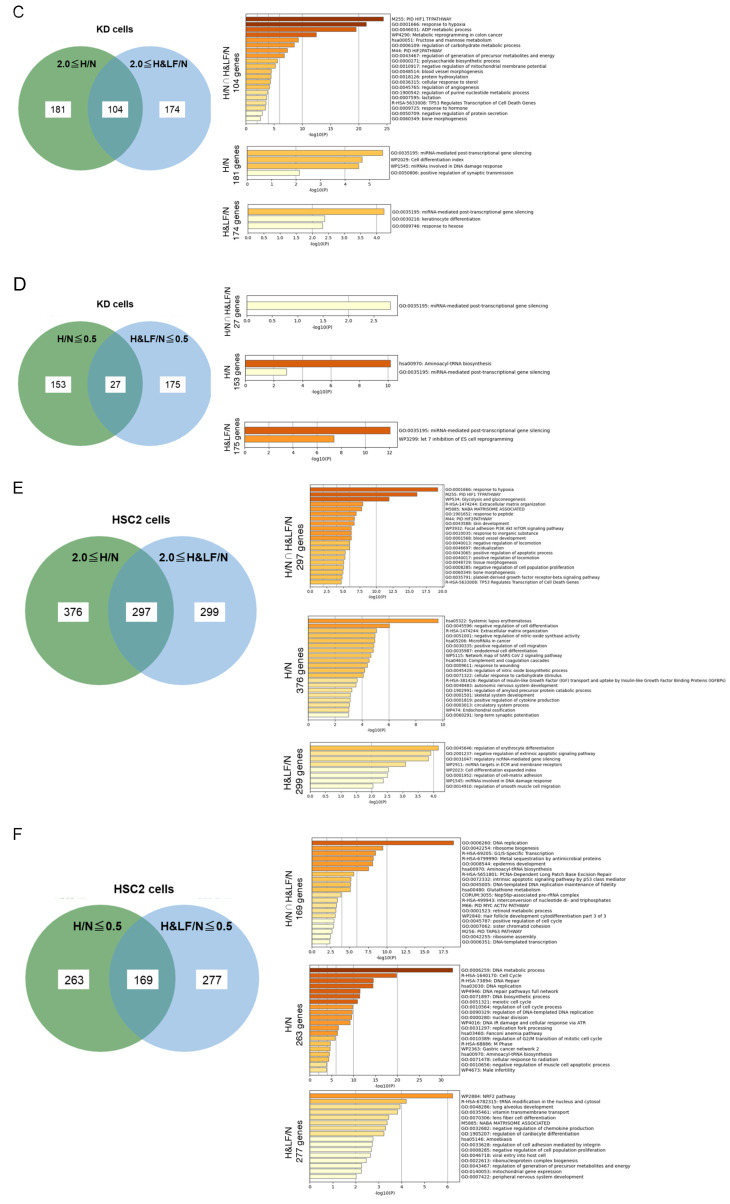
Lactoferrin regulates multiple signaling pathways including apoptosis and NRF2 antioxidant responses. Comprehensive gene expression analysis followed by gene set enrichment analysis (GSEA) was performed on KD and HSC2 cells. (**A**) Gene sets with altered expression under hypoxic conditions were compared. (**Top**) Gene sets commonly altered; Middle: Gene sets altered in KD cells but not in HSC2 cells; (**Bottom**) Gene sets altered in HSC2 cells but not in KD cells. (**B**) Gene sets with altered expression by lactoferrin (LF) treatment were compared. (**Top**) Gene sets commonly altered; Middle: Gene sets altered in KD cells but not in HSC2 cells; (**Bottom**) Gene sets altered in HSC2 cells but not in KD cells. Gene sets upregulated (**C**) and downregulated (**D**) expression with lactoferrin treatment under hypoxic conditions in KD cells. Gene sets upregulated (**E**) and downregulated (**F**) expression with lactoferrin treatment under hypoxic conditions in HSC2 cells.

To clarify the molecular signals altered by LF treatment in irradiated KD and HSC2 cells, a comprehensive gene expression analysis followed by gene set enrichment analysis (GSEA) was performed. Gene sets with altered expression under hypoxic conditions were compared in KD and HSC2 cells (Figure 4A and Figure S2A). The GSEA revealed that genes involved in HIF-1-regulated hypoxic responses were activated in both cell lines. Genes related to tRNA biosynthesis, non-coding RNA-mediated post-translational gene silencing, and ADP metabolic processes were altered in KD cells, whereas genes involved in DNA replication, homology-directed repair, the mitotic cell cycle, nuclear DNA replication, and PCNA-dependent long-patch base excision repair were altered in HSC2 cells.

LF treatment commonly regulates genes related to miRNA-mediated gene silencing by inhibiting translation and post-transcription gene silencing in both cells (Figure 4B and Figure S2B). Specific genes associated with miRNA-mediated post-transcription gene silencing, let7 inhibition of ES cell reprogramming, and aminoacyl-tRNA biosynthesis in KD cells, and miRNA-mediated post-transcription gene silencing, aminoacyl-tRNA biosynthesis, interferon alpha/beta signaling, miRNA-mediated gene silencing by inhibition of translation, regulation of cytokine production, cell migration, angiogenesis, pattern recognition receptor signals, and extrinsic apoptosis signals in HSC2 cells, were altered, respectively. Further comparative analyses of the different combinations were performed where possible (Figure S2C,D). For example, LF treatment upregulated the expression of genes related to miRNA-mediated post-transcription gene silencing, keratinocyte differentiation, and the response to hexose in KD cells under hypoxic conditions (Figure 4C), but downregulated miRNA-mediated post-transcription gene silencing and let7 inhibition of ES cell reprograming genes (Figure 4D). On the other hand, LF treatment upregulated expressions of genes related to the regulation of erythrocyte differentiation, the negative regulation of extrinsic apoptotic signaling, the regulation of ncRNA-mediated gene silencing, miRNA targets in ECM and membrane receptors, and the cell differentiation expanded index in HSC2 cells (Figure 4E). In addition, the NRF2 pathway, tRNA modification in the nucleus and cytosol, lung alveolus development, vitamin transmembrane transport, lens fiber cell differentiation, the matrisome, and the negative regulation of chemokine production were downregulated in HSC2 cells with LF treatment under hypoxic conditions (Figure 4F).

### 3.5. Lactoferrin Regulates Gene Expressions of Apoptotic, NRF2 Signal Pathway, and DNA Damage Responses

**Figure 5:**
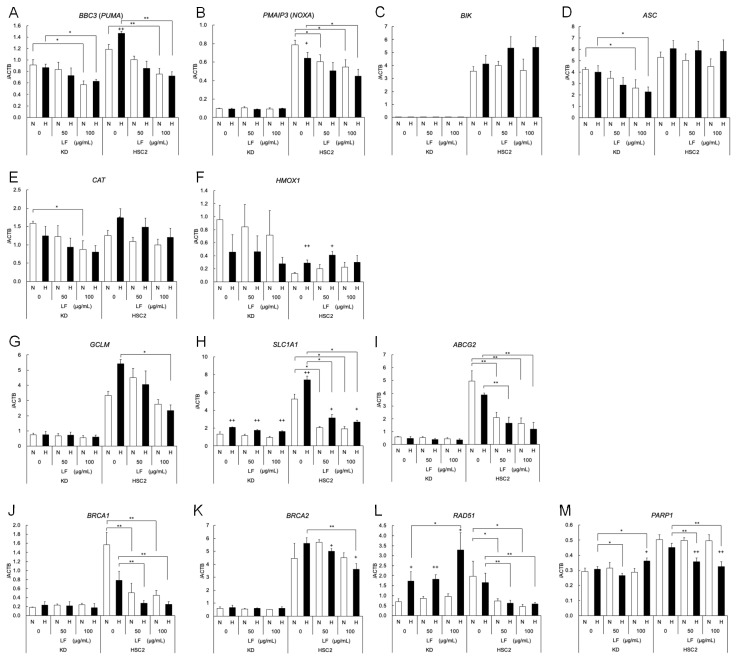
Lactoferrin regulates the gene expression of apoptosis, NRF2 signaling pathway, and DNA damage response genes. Effects of LF treatment on the expression of (**A**–**D**) apoptosis, (**E**–**I**) NRF2 signaling pathway, and (**J**–**M**) DNA damage response genes in KD and HSC2 cells under normoxic (N) and hypoxic (H) conditions for 24 h were evaluated by quantitative RT-PCR. Relative gene expression levels were calculated using *ACTB* expression as the denominator for each cell line (*n* = 3). The mean and SD are shown for all quantitative values. Statistical significance is indicated by + *p* < 0.05 and ++ *p* < 0.01 (N vs. H), * *p* < 0.05, and ** *p* < 0.01 (paired samples indicated).

Previously, we demonstrated that the expression levels of anti-apoptotic *BCL2* were decreased only in HSC2 cells with LF treatment, but not in KD cells. Those of pro-apoptotic *BAX* were slightly decreased by LF treatment in both cells. Since RNA-seq and GSEA suggested that LF regulates extrinsic apoptotic signaling, the expression levels of apoptosis-related genes in both KD and HSC2 cells were further evaluated using quantitative RT-PCR (Figure 5A–D). The expression of pro-apoptotic genes, *BBC3* (*PUMA*), *PMAIP3* (*NOXA*), and *BIK* was significantly higher in HSC2 cells. In particular, the expression levels of *PMAIP3* and *BIK* were very low in KD cells (Figure 5A–C). LF treatment decreased the expression levels of *BBC3* in both cells, and that of *PMAIP3* in HSC2 cells. The expression of *BIK* did not respond to LF treatment. Strikingly, the expression of extrinsic pro-apoptotic *ASC* was significantly decreased by LF treatment only in KD cells and not in HSC2 cells (Figure 5D).

Since RNA-seq also suggested that LF downregulated the hypoxic expression of genes related to the NRF2 pathway and oxidative stress response, these expression levels were also evaluated using quantitative RT-PCR (Figure 5E–I). The expression levels of *CAT* were slightly decreased in both cell lines (Figure 5E). Those of *HMOX1* were significantly increased in hypoxic HSC2 cells, but decreased in KD cells, although it was not significant (Figure 5F). LF treatment did not seem to affect their expression in either cell line. The expression levels of NRF2 targets, *GCLM*, *SLC1A1*, and *ABCG2*, were significantly higher in HSC2 cells and were very low in KD cells (Figure 5G–I). *SLC1A1* expression was significantly increased under hypoxic conditions in both cell lines (Figure 5H). Strikingly, the expression levels of these genes were significantly decreased by LF treatment only in HSC2 cells and not in KD cells. Further expression of oxidative stress-induced DNA damage repair genes was evaluated (Figure 5J–M). The expression levels of *BRCA1*, *BRCA2*, *RAD51*, and *PARP1* were significantly higher in HSC2 cells, and were decreased by LF treatment in HSC2 cells. However, levels of *RAD51* and *PARP1* were slightly increased in KD cells.

### 3.6. Dual Role of Lactoferrin in Modulating Radiation Responses Under Hypoxic Conditions Through Extrinsic Apoptosis and NRF2

**Figure 6:**
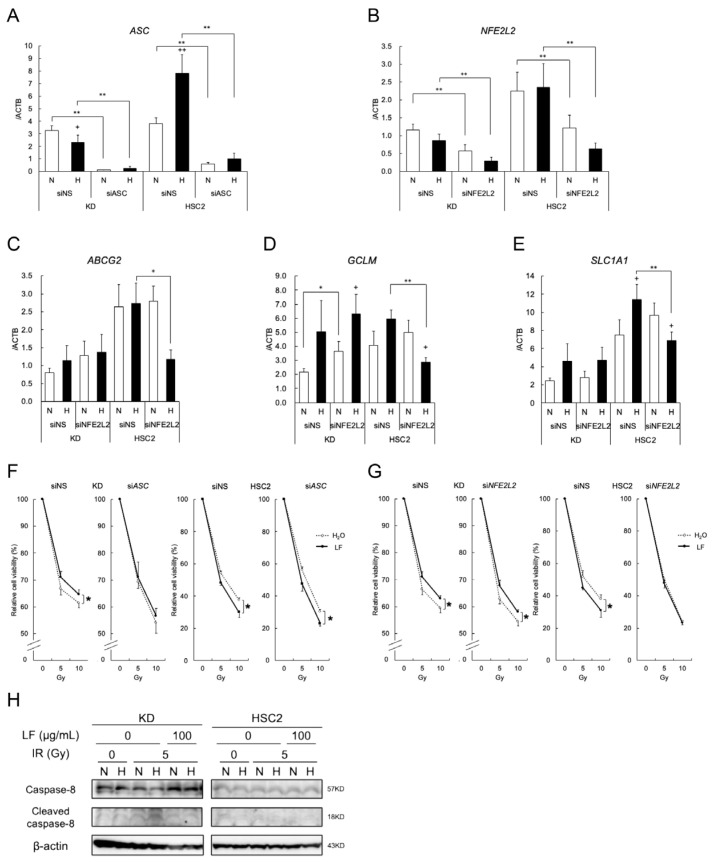
Dual role of lactoferrin in modulating radiation responses under hypoxic conditions through extrinsic apoptosis and NRF2. (**A**,**B**) KD and HSC2 cells were transfected with siRNA for non-specific (siNS), *PYCARD* (siASC), or *NFE2L2* (siNRF2) siRNA, and were incubated under normoxic (N) and hypoxic (H) conditions for 24 h. Expression of *PYCARD* (*ASC*) or *NFE2L2* (*NRF2*) was evaluated using quantitative RT-PCR. (**C**–**E**) Expression of NRF2 target genes in NRF2-knockdown KD and HSC2 cells was evaluated using quantitative RT-PCR. (**F**,**G**) To evaluate the response to radiation, cells were irradiated and treated with LF after siRNA transfection, then further incubated under hypoxic conditions for 72 h. Cell proliferation capacity was evaluated using the MTT assay. (**H**) Radiation-induced caspase-8 activation in KD and HSC2 cells with/without lactoferrin treatment under normoxic or hypoxic conditions was analyzed using immunoblots. β-actin was used as an internal loading control. Representative images of three independent experiments are shown. Values are mean and SD (*n* = 3). Statistical significance is indicated by + *p* < 0.05 (N vs. H) and ++ *p* < 0.01 (N vs. H), * *p* < 0.05, and ** *p* < 0.01 (paired samples indicated).

Given the importance of ASC in KD cells and NRF2 in HSC2 cells, knockdown experiments were performed. RT-PCR confirmed the effective knockdown of *ASC* and *NFE2L2* (*NRF2*) in KD and HSC2 cells (Figure 6A,B). Furthermore, knockdown of *NFE2L2* in HSC2 cells showed a significant reduction in NRF2 target gene expression under hypoxic conditions (Figure 6C–E). The MTT assays strikingly demonstrated that the protective effects of LF against irradiation were abolished by the knockdown of *ASC* in KD cells under hypoxic conditions, but this was not observed in HSC2 cells (Figure 6F). On the other hand, knockdown of *NFE2L2* eliminated the sensitizing effects of LF on irradiation in HSC2 cells under hypoxic conditions, but this was not the case in KD cells (Figure 6G). Immunoblot analyses further suggested that cleaved caspase-8 was increased with irradiation in KD cells and also increased under hypoxic conditions (Figure 6H). LF treatment further decreased cleaved caspase-8 in KD cells under hypoxic conditions, but these effects were not observed in HSC2 cells.

## 4. Discussion

In this study, we have demonstrated an intriguing dual role for lactoferrin in modulating radiation responses in a cell-specific manner under hypoxic conditions, providing valuable insights into its potential use in radiotherapy. The observed differential effects of lactoferrin on cancer (HSC2) and non-cancer (KD) cells may be largely attributed to its distinct influence on reactive oxygen species (ROS) production and the downstream regulation of apoptosis and antioxidant signaling pathways. These findings suggest a sophisticated interaction between hypoxia, lactoferrin, and cellular redox states that could be exploited to improve therapeutic outcomes in cancer treatment.

**Figure 7:**
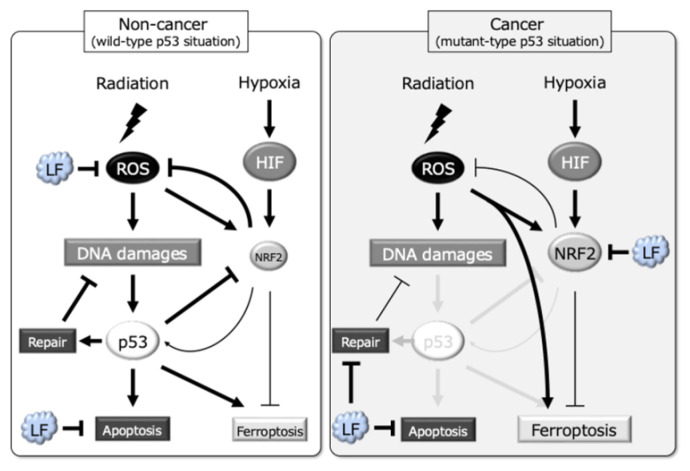
Schematic representation of the dual role of lactoferrin in modulating radiation responses under hypoxic conditions through apoptosis and ferroptosis in non-cancer and cancer cells.

The role of ROS in radiotherapy is complex because ROS generated by radiation induce oxidative stress, leading to DNA damage and apoptosis in cancer cells. However, hypoxic conditions commonly found in solid tumors are known to reduce ROS production, which impairs radiation efficacy and leads to radioresistance by limiting oxidative stress and subsequent DNA damage [5,6]. Our study demonstrates that lactoferrin differentially modulates ROS production in cancer and non-cancer cells under hypoxia: it reduces ROS in KD cells, providing potential protection, while increasing ROS in HSC2 cells, thereby amplifying radiation-induced oxidative damage (Figure 7). This dual role is consistent with previous findings that lactoferrin exerts selective cytotoxicity across cancer cell types by affecting their redox environment, thereby enhancing or attenuating radiation effects depending on the cellular context [16].

The effect of lactoferrin on ROS levels also influences the DNA damage response, as indicated by changes in γH2AX foci, a marker of double-strand breaks (DSBs). The reduction of γH2AX foci in KD cells with lactoferrin treatment suggests that it may enhance DNA repair pathways or mitigate oxidative stress via NRF2-mediated antioxidant responses, consistent with the roles of NRF2 in protecting cells from oxidative damage [37,38]. Conversely, the increase in DSBs in HSC2 cells with lactoferrin treatment may be due to increased ROS that promote apoptosis in the context of weakened antioxidant defenses, as lactoferrin specifically downregulates NRF2 pathway genes in cancer cells under hypoxic conditions. In addition, lactoferrin treatment reduced the expression levels of a number of DNA repair genes in HSC2 cells (Figure 7). One possible mechanism for these differential responses is the difference in p53-dependent cellular responses, which has been reported previously [29]. In a p53-mutated state (in HSC2 cells), the NRF2 pathway is highly activated, as shown in this study, resulting in NRF2-dependent cell survival [39]. This selective impairment of antioxidant defenses and DNA damage responses could enhance radiation sensitivity in cancer cells while sparing healthy cells, underscoring the potential of lactoferrin treatment in cancer-selective radiosensitization. In addition, the role of lactoferrin in modulating extrinsic apoptosis pathways under hypoxic conditions appears to be critical for this differential effect. Lactoferrin treatment appears to inhibit intrinsic apoptosis signal in both cells, as indicated by decreased levels of *BBC3* (*PUMA*), *PMAIP3* (*NOXA*), and *BAX*. However, the selective downregulation of extrinsic pro-apoptotic *ASC* in KD cells with lactoferrin treatment suggests a protective mechanism against radiation-induced cell death. These findings are further supported by knockdown experiments in which depletion of *ASC* in KD cells and *NFE2L2* (*NRF2*) in HSC2 cells abolished the protective and sensitizing effects of lactoferrin, respectively (Figure 7). These findings also support previous research suggesting the ability of lactoferrin to modulate both pro-apoptotic and pro-survival signaling, depending on cellular stressors and environmental conditions [40,41,42,43,44].

Ferroptosis is a newly recognized iron-dependent form of cell death induced by the accumulation of lipid peroxides, leading to redox imbalance, and it is now known as a critical driver in numerous pathological processes, including neurodegeneration, ischemia-reperfusion injury, and cancer progression. It has been reported that the tumor-suppressive functions of ferroptosis in this context involve the regulation of reactive oxygen species production via p53 and *SLC7A11* [45,46]. We have also previously demonstrated that LF increased the expression of the ferroptosis-promoting *ACSL4* gene in cells with mutant-type p53 under hypoxic conditions. In this study, we further observed the downregulation of NRF2 target genes, namely *ABCG2*, *GCLM*, and *SLC1A1*, which are involved in ferroptosis regulation, only in HSC2 cancer cells (Figure 7). Since NRF2 has been shown to regulate radiation-induced ferroptosis in lung cancer [47], our study strongly suggests that LF suppresses antioxidant capacity and promotes ferroptosis by regulating NRF2 signaling in cancer cells.

Although this study clearly demonstrated the important role of lactoferrin-mediated control of ROS production in the response to radiation, the detailed mechanisms of lactoferrin’s effects on ROS production and the antioxidant NRF2 pathway remain to be determined. Furthermore, although knockdown experiments in this study clearly indicate the significant role of HIF-1α in the effects of lactoferrin on radiation responses, the detailed mechanisms remain unclear. A previous report suggested that choroidal neovascularization formation in mice was suppressed by oral administration of lactoferrin via inactivation of HIF-1α in the retinal pigment epithelium and neural retina [48]. However, changes in HIF-1 downstream signaling with lactoferrin treatment were not observed in this study. Further studies are needed to clarify these detailed mechanisms.

## 5. Conclusions

In conclusion, the influence of lactoferrin on ROS, DNA damage responses, and apoptosis pathways in hypoxic cancer and non-cancer cells highlights its potential as a dual-function therapeutic agent. Lactoferrin may act as a radiosensitizer in hypoxic tumors by increasing oxidative stress and DNA damage, while acting as a radioprotector in normal tissues by maintaining redox homeostasis and preventing apoptosis. Further exploration of the interactions between lactoferrin, hypoxic signaling, and antioxidant defenses may establish its role as an adjunct in radiotherapy protocols, improving therapeutic outcomes with reduced toxicity in healthy tissues.

## Data Availability

The original contributions presented in this study are available in the article/Supplementary Materials. For further information, please contact the corresponding author.

## Supplementary Materials

The following supporting information can be downloaded at: https://www.mdpi.com/article/10.3390/antiox14010001/s1, Figure S1: Effects of lactoferrin on radiation responses under hypoxic conditions; Figure S2: Lactoferrin regulates multiple signaling pathways.
