# Supplementary material for: Lactoferrin Modulates Radiation Response Under Hypoxic Conditions, Possibly Through the Regulation of ROS Production in a Cell Type-Specific Manner
Source: Antioxidants (Basel). 2024 Dec 24;14(1):1. doi: 10.3390/antiox14010001 (PMC11762174; doi:10.3390/antiox14010001)
Supplement: Supplementary file 1 [file antioxidants-14-00001-s001.zip › antioxidants-3376799-supplementary.pdf]

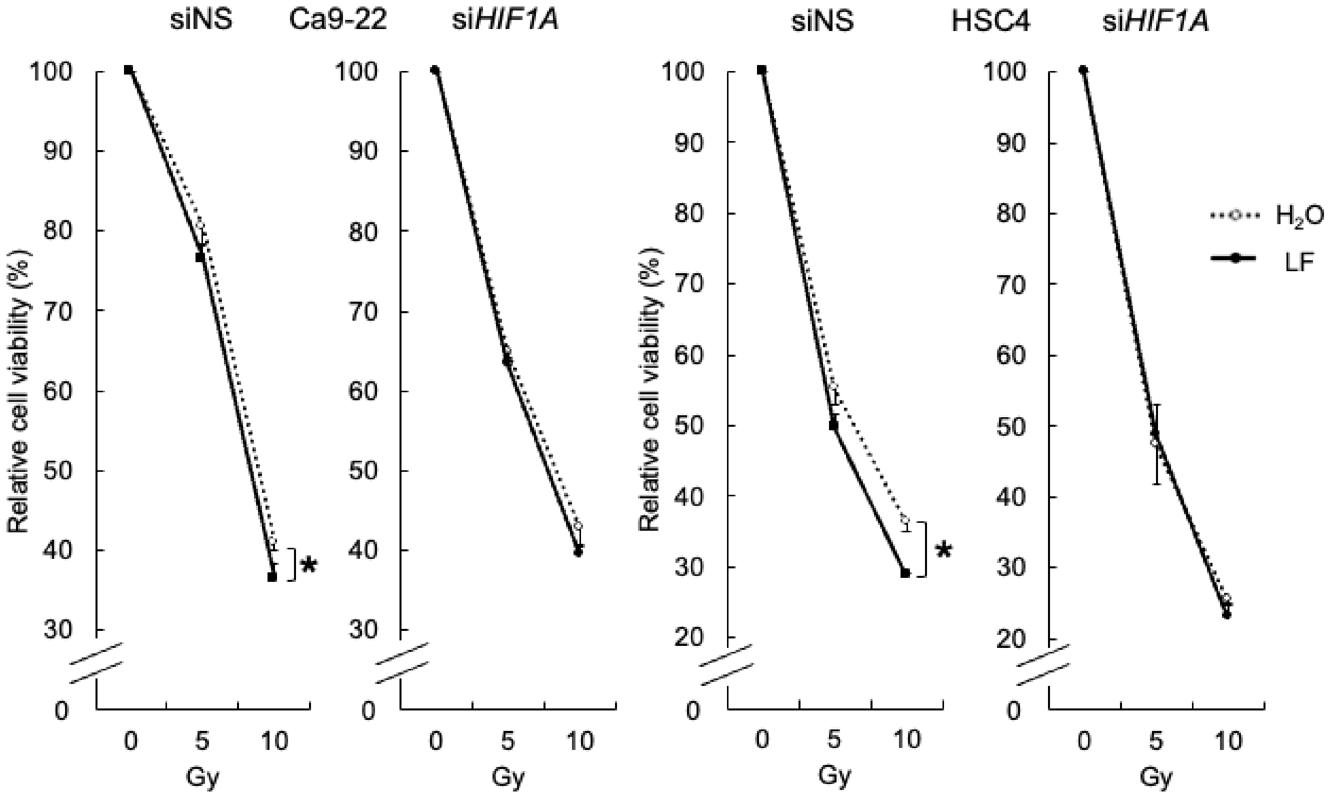

Figure S1. Effects of lactoferrin on radiation responses under hypoxic conditions.
Cells were transfected with non-specific control (siNS) or siHIF1A 24 hours after seeding, and further incubated for 24 hours before irradiation. Lactoferrin was added to the cell culture medium immediately after irradiation. After further incubation under hypoxic conditions for 72 hours, the relative cell viability of Ca9-22 and HSC4 cells after γ-ray irradiation (0, 5, or 10 Gy) was evaluated by the MTT assay. Values are mean and SD (n = 3). Statistical significance is indicated by $*P < 0.05$.

A

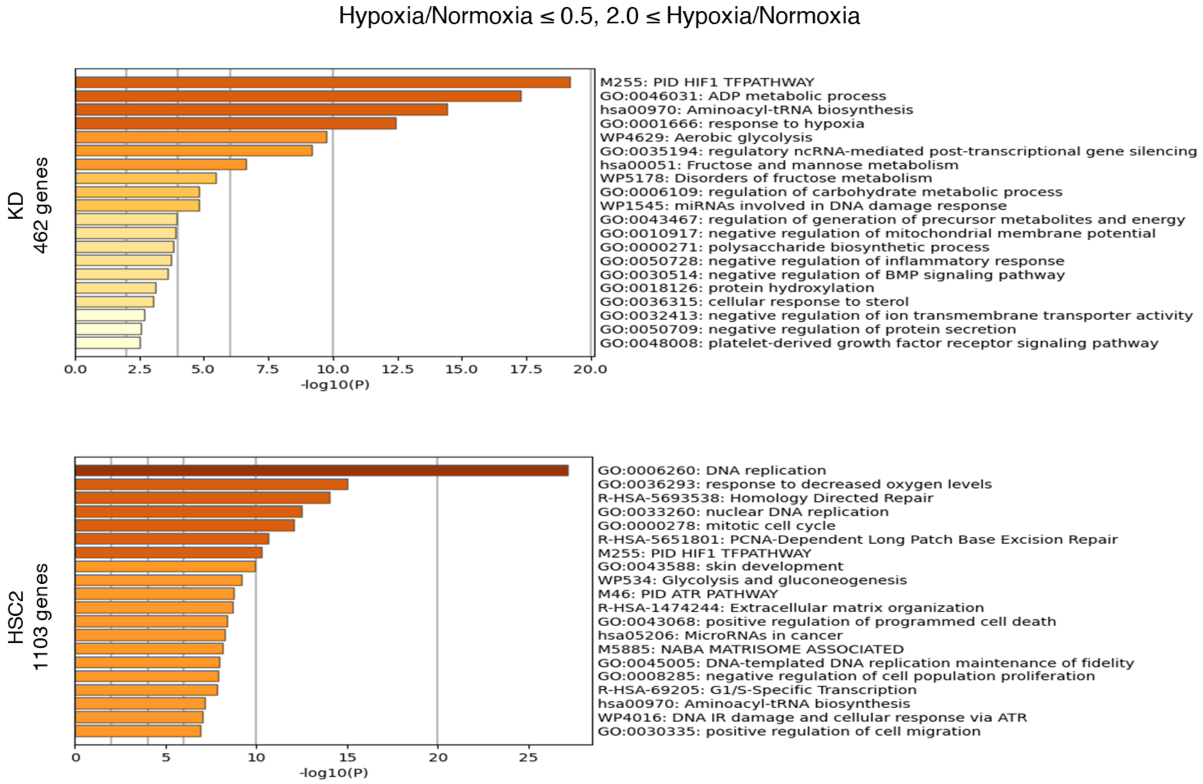
Hypoxia/Normoxia ≤ 0.5, 2.0 ≤ Hypoxia/Normoxia
KD
462 genes
M255: PID HIF1 TFPATHWAY
GO:0046031: ADP metabolic process
hsa00970: Aminoacyl-tRNA biosynthesis
GO:0001666: response to hypoxia
WP4629: Aerobic glycolysis
GO:0035194: regulatory ncRNA-mediated post-transcriptional gene silencing
hsa00051: Fructose and mannose metabolism
WP5178: Disorders of fructose metabolism
GO:0006109: regulation of carbohydrate metabolic process
WP1545: miRNAs involved in DNA damage response
GO:0043467: regulation of generation of precursor metabolites and energy
GO:0010917: negative regulation of mitochondrial membrane potential
GO:0000271: polysaccharide biosynthetic process
GO:0050728: negative regulation of inflammatory response
GO:0030514: negative regulation of BMP signaling pathway
GO:0018126: protein hydroxylation
GO:0036315: cellular response to sterol
GO:0032413: negative regulation of ion transmembrane transporter activity
GO:0050709: negative regulation of protein secretion
GO:0048008: platelet-derived growth factor receptor signaling pathway
0.0 2.5 5.0 7.5 10.0 12.5 15.0 17.5 20.0
-log10(P)
HSC2
1103 genes
GO:0006260: DNA replication
GO:0036293: response to decreased oxygen levels
R-HSA-5693538: Homology Directed Repair
GO:0033260: nuclear DNA replication
GO:0000278: mitotic cell cycle
R-HSA-5651801: PCNA-Dependent Long Patch Base Excision Repair
M255: PID HIF1 TFPATHWAY
GO:0043588: skin development
WP534: Glycolysis and gluconeogenesis
M46: PID ATR PATHWAY
R-HSA-1474244: Extracellular matrix organization
GO:0043068: positive regulation of programmed cell death
hsa05206: MicroRNAs in cancer
M5885: NABA MATRISOME ASSOCIATED
GO:0045005: DNA-templated DNA replication maintenance of fidelity
GO:0008285: negative regulation of cell population proliferation
R-HSA-69205: G1/S-Specific Transcription
hsa00970: Aminoacyl-tRNA biosynthesis
WP4016: DNA IR damage and cellular response via ATR
GO:0030335: positive regulation of cell migration
0 5 10 15 20 25
-log10(P)

B

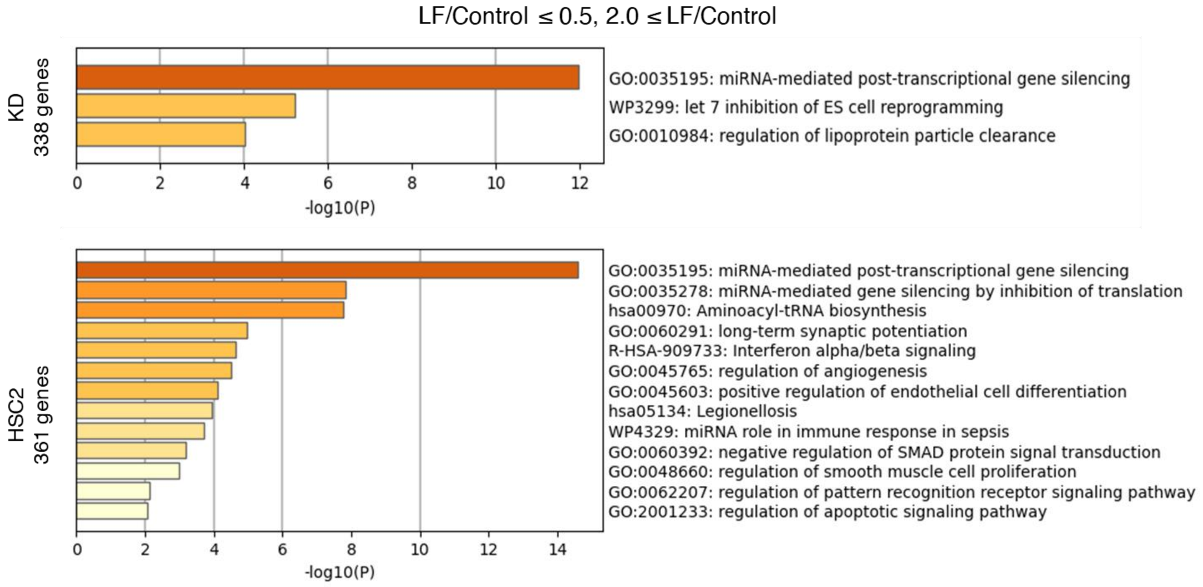
LF/Control ≤ 0.5, 2.0 ≤ LF/Control
KD
338 genes
GO:0035195: miRNA-mediated post-transcriptional gene silencing
WP3299: let 7 inhibition of ES cell reprogramming
GO:0010984: regulation of lipoprotein particle clearance
0 2 4 6 8 10 12
-log10(P)
HSC2
361 genes
GO:0035195: miRNA-mediated post-transcriptional gene silencing
GO:0035278: miRNA-mediated gene silencing by inhibition of translation
hsa00970: Aminoacyl-tRNA biosynthesis
GO:0060291: long-term synaptic potentiation
R-HSA-909733: Interferon alpha/beta signaling
GO:0045765: regulation of angiogenesis
GO:0045603: positive regulation of endothelial cell differentiation
hsa05134: Legionellosis
WP4329: miRNA role in immune response in sepsis
GO:0060392: negative regulation of SMAD protein signal transduction
GO:0048660: regulation of smooth muscle cell proliferation
GO:0062207: regulation of pattern recognition receptor signaling pathway
GO:2001233: regulation of apoptotic signaling pathway
0 2 4 6 8 10 12 14
-log10(P)

C

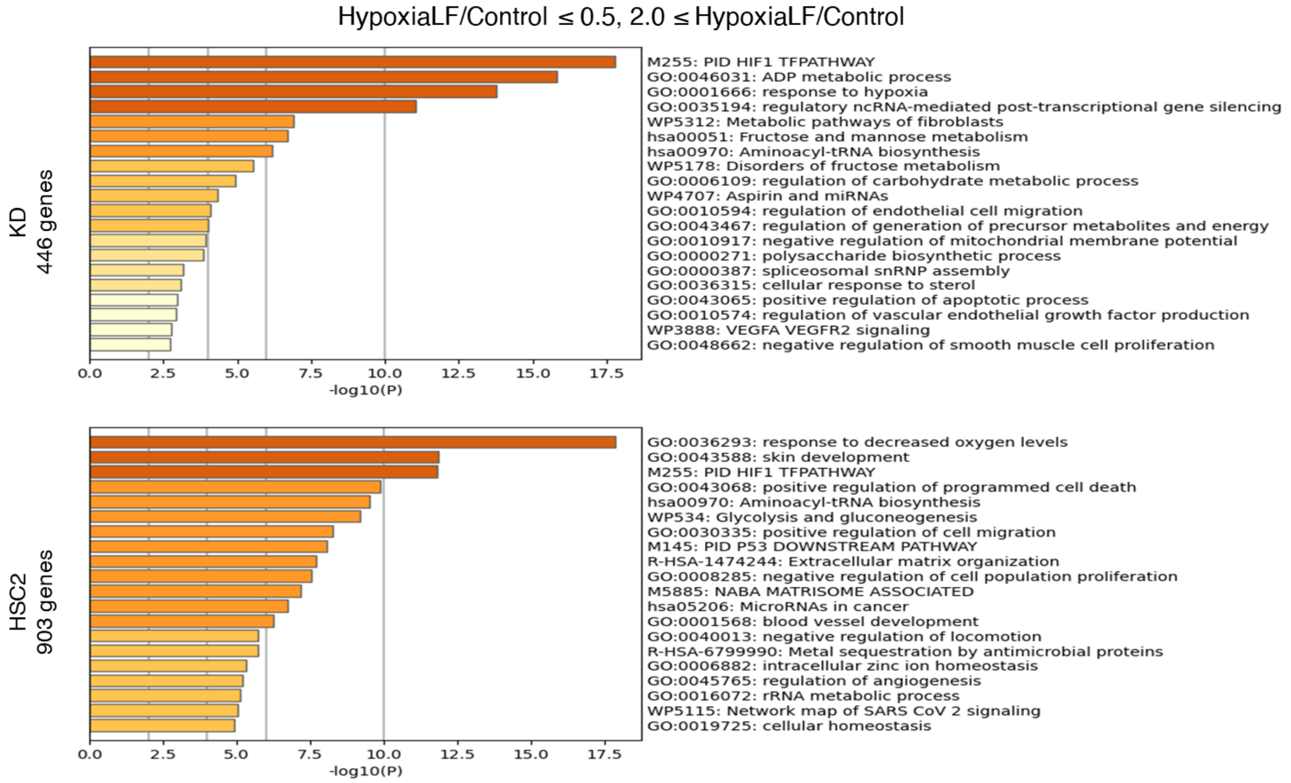

D

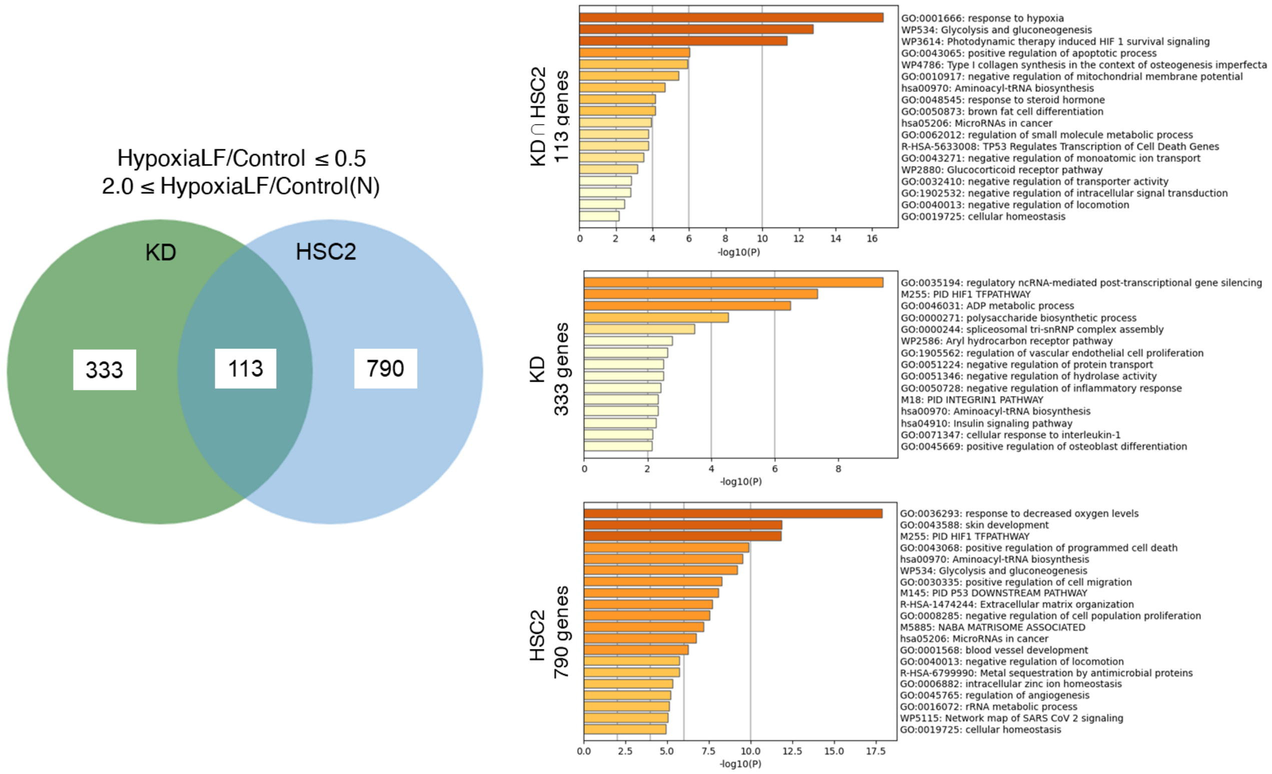

Figure S2. Lactoferrin regulates multiple signaling pathways.
Comprehensive gene expression analysis followed by gene set enrichment analysis (GSEA) were performed. (A) Gene sets with altered expression under hypoxic conditions were analyzed. Top: Gene sets altered in KD cells; Bottom: Gene sets altered in HSC2 cells. (B) Gene sets whose expression was altered by lactoferrin (LF) treatment were analyzed. Top: Gene sets altered in KD cells; Bottom: Gene sets altered in HSC2 cells. (C) Gene sets altered

in expression with LF treatment under hypoxic conditions were analyzed. Top: Gene sets altered in KD cells; Bottom: Gene sets altered in HSC2 cells. (D) Gene sets altered in expression with LF treatment under hypoxic conditions were compared between KD and HSC2 cells. Top: Gene sets commonly altered; Middle: Gene sets altered in KD cells but not in HSC2 cells; Bottom: Gene sets altered in HSC2 cells but not in KD cells.
